# Should We Perform Semen Analysis, DNA Fragmentation, and Hypo-osmotic Swelling Tests together?

**Published:** 2016-11-13

**Authors:** Samaneh Hasanzadeh Keshteli, Mir Mehrdad Farsi, Soraya Khafri

**Affiliations:** 1 *Department of Anatomical Sciences, School of Medicine, Babol University of Medical Sciences, Babol, Iran.*; 2 *Department of Biostatistics and Epidemiology, Babol University of Medical Sciences, Babol, Iran.*

**Keywords:** Semen analysis, morphology, DNA fragmentation, hypo-osmotic swelling test, IVF

## Abstract

Semen analysis, sperm DNA fragmentation (SDF) and hypo-osmotic swelling test (HOST) are usually performed for the evaluation of sperm fertilizing ability. There are some debates over the necessity of SDF and HOST incorporation in male infertility work-up.Semen of 77 men was evaluated by SDF and HOST through three semen analyses. Sperm parameters were arranged into different categories: <5%, 5-15%, >15% for normal morphology; <50%, 50-70%, >70 % for motility; and <10, 10-20, 21-34, 35-50, >50 million/ml for concentration. SDF analysis was performed and values under 30% were assumed to be normal. Normal range of HOST was considered to be >60%.Only normal sperm morphology had significant relationship with DF rate (P<0.001). Normal morphology, motility, and concentration of sperms had significant relationship with HOST (P<0.001, 0.05, and <0.003,respectively). There was a significant negative correlation between sperm morphology and DF rate. The correlations between sperm parameters and percentage of HOST were significantly positive (r: 0.44, 0.19, and 0.32 for morphology, motility, and concentration, respectively). Receiver operating characteristic curve (ROC) showed that sperm morphology is a strong predictor of the rate of DF and HOST (accuracy: 0.74‚ and 0.81, respectively). The best sperm morphology cut off point for DF and HOST rate prediction was 4.5% and 5.5%, respectively.Sperm morphology had significant correlation with DF rate and HOST and is supposed to be a predictor for these tests. Performing these three tests collectively for evaluation of semen samples would not be necessarily required in all cases.

Sperm and oocyte are the main actors in creating and continuity of generations. Any defects in structure and function of these cells may lead to failure of fertilization, the fundamental process of beginning of life. It has been shown that male factor is the major problem in half of infertile couples (1), and in the field of assisted reproductive technique (ART), evaluation of sperm quality might be of great value (2). Routine semen analysis is not a perfect test for this purpose; so sperm DNA fragmentation (SDF) and hypo-osmotic swelling test (HOST) have been proposed as more valuable and reliable tests (3-5).

Sperm DNA integrity for correct transmission of paternal genetic information (6) is the basis for SDF assay. This test is related to the presence of breaks in one or two strands of DNA in human spermatozoa (7,8). Defective chromatin condensa-tion during spermiogenesis, apoptosis during spermatogenesis and oxidative stress (9) are major mechanisms leading to DNA damage. Although sperm DNA damage may be transmitted to the next generation, it has been reported that level of DNA fragmentation (DF) did not predict pregnancy outcome in intra cytoplasmic sperm injection (ICSI) cycles (10). Functional integrity of sperm membrane as a barrier between intra and extra cellular spaces and a sign for DNA integrity (11) can be assessed by hypo-osmotic swelling test (HOST). HOST is a common, low cost, simple and reliable test (12, 13) which may reveal functional ability of sperms including acrosome reaction, sperm capacitation, and the binding of spermatozoa to the oocyte surface (14). In viable spermatozoa, water (fluid) passes across the sperm membrane and causes swelling in the sperm tail (15). Different tail patterns may happen from (a) to (g) according to World Health Organization (WHO) where (a) is a dead spermatozoa and without tail changing. (b)– (g) have various types of tail changes. In case of as the nozoospermia and testicular immotile sper-matozoa, HOST is useful for distinguishing of dead spermatozoa from viable immotile sperm (16, 17).

There are dissimilarities in reports of relationship among routine semen analysis, SDF, and HOST. Negative correlation )^18^, ^19^(, and no correlation between sperm parameters and SDF (20, 21) have been reported. Also, the reported correlations are different between sperm paramete-rs and HOST values (22-27). In Stanger's report, there was a strong correlation among sperm parameters, DF rate, and HOST value (28). In spite of these dissimilarities, existence of the correlations and predictive values among these three tests may lead to lack of necessity for performing these tests together in IVF labs. To the best of our knowledge, this is the first investigation which statistically analyzes the relationship of three functional sperm tests in the lab. In this case, we investigated the distribution of levels of DF and HOST values in different sperm parameter categories, the correla-tion between sperm parameters and DF, and sperm membrane response in hypo-osmolar condition. Sperm parameters as predictors of DF rate and HOST were analyzed as well.

## Materials and methods

**Patients and semen analysis**

This cross-sectional study was conducted on 77 infertile couples (age range, 24–50 years) who were referred to IVF lab of Mehregan Hospital, Babol, Iran. Sample size was determined according to a similar article with confidence level of 95% and power of 80% (28). Ethics Committee center approved the study. Semen sample was collected with the aid of spouses after 3-5 days of abstinence. After complete liquefaction, each sample was aliquoted for three separate portions. All samples were observed under light microscope. Sperm concentration and motility were assessed according to WHOguidelines (29). Sperm morphology was assessed according to Tygerberg strict criteria (30) after Papanicolaou staining procedure. A second aliquot of each sample was prepared immediately for DF rate assessment, and a third aliquot for HOST (12).

**Assessment of sperm DNA fragmentation (SDF)**

SDF analysis was performed using Halosperm kit (Parque Tecnológico de Madrid, Spain). Each sperm sample was diluted in culture media to a maximum of 20 million sperm per ml. Fifty µl of each semen aliquot was mixed with 100 µl liquefied agarose. Then 50 µl of semen-agarose mixture was placed on the slide. After DNA denaturation of fragmented sperms, nuclear proteins were removed by adding lysis solution in Halosperm kit (Parque Tecnológico de Madrid Spain). Finally, dehydration and staining by colors in Halosperm kit (Parque Tecnológico de Madrid Spain) was performed for each slide. At least 300 spermatozoa were assessed. Sperm heads with large hollows of spreading DNA loops emerging from a central core were assessed as absence of massive DNA breakage. Sperms with large halos (thicknesses that were similar or larger than the length of the smallest diameter of the core) and sperm with medium sized halos (thickness greater than 1/3 of the smallest diameter of the core and less than the smallest diameter of the core) were classified as spermatozoa without DF. Sperms with a small halo head (equal or less than 1/3 of the smallest diameter of sperm head) or no halo head, were assessed as DF spermatozoa. Semen samples with SDF rate <30% were considered as low fragmentation group (LFG) and samples with SDF rate ≥30% as high fragmentation group (HFG).

**Assessment of vitality (hypo-osmotic swelling test)**

HOST was performed on an aliquot of each semen sample. 50µl of semen sample diluted with 100 µl of hypo-osmotic swelling solution (50 % Hams+50 % purified water) was left to incubate at 25-37°C for 5 min. At least 100 spermatozoa with different patterns of HOST from (a) to (g), were assessed according to WHO by light microscopy. Semen samples with ≥ 60% positive swelling tail reaction were considered as normal while those with less than 60% positive swelling tail reaction were considered as abnormal.

**Statistical analyzes**

Statistical analyses were performed by SPSS software (version 23, SPSS Inc, Chicago, IL, USA). Categorical data were analyzed by chi-square test. The cut off values were determined by ROC analysis and sensitivity and specificity were calculated. A p*-*value of less than 0.05 was considered as statistically significant.

## Results

**Conventional semen analysis**

**Fig. 1:**
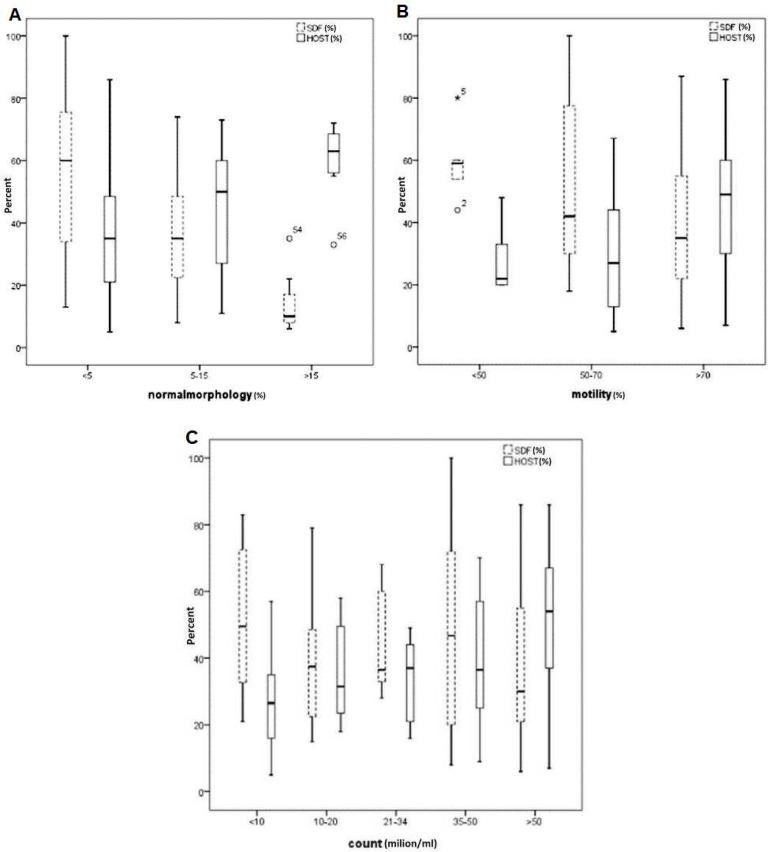
Distribution of DF rate and hypo-osmotic swelling score in sperm parameters categories. A: categories of sperm morphology (<5% , 5-15% , >15%);B: motility (<50% , 50-70% , >70 % ); and C: concentration (<10, 10-20, 21-34,35-50, >50 million/m

The mean (±SD) of sperm concentration was 48.71 (±34.65); for sperm motility, progress and normal sperm morphology were 78.05 (±15), 75.71(±9.6) and 7.31 (±6.30), respectively. The distribution of levels of DF and HOST scores in sperm parameters categories are illustrated in box plot diagrams (Fig. 1). Half of the samples in two categories of sperm morphology i.e. <5% and 5-15%, had the DF rate above 30%. The distribution of normal HOST score in category of >15% normal morphology was higher in comparison to others (Fig. 1A).

**Sperm DNA fragmentation assessment**

**Fig. 2:**
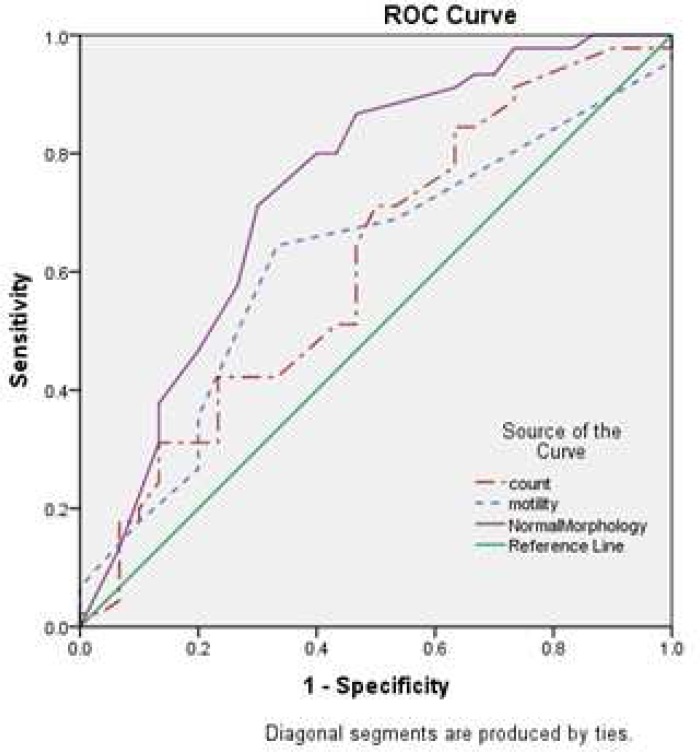
Receiver operating characteristic (ROC) curve analysis for the prediction of SDF by sperm parameters. The area under the curve (AUC) for normal morphology, motility and concentration was 0.74, 0.61 and 0.61, respectively

The mean (± SD) of SDF was 43.52 (±23.96). There was a high significant negative correlation between sperm normal morphology and DF (r=-0.6; P≤ 0.001, Table 1). The category of>15%sperm with normal morphology had a lower DF than the other categories (Fig. 1A). The relationships between sperm motility and concentration with DF were not statistically significant (P= 0.1, P= 0.9, Table 1).Sperm parameters as predictors of DF were evaluated by ROC curve (Fig. 2).

**Hypo-** **osmoticswelling test**

**Fig. 3:**
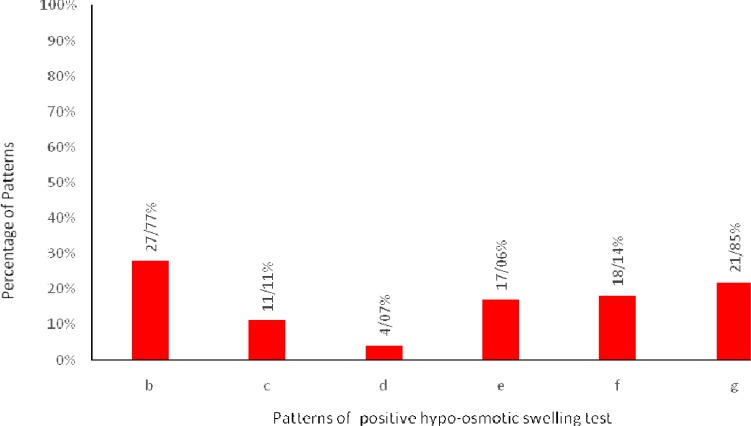
Frequency of different patterns of positive HOST according to WHO (magnification at 100 x

The mean (±SD) of HOST score was 41.49 (± 19.37). Distribution of different patterns of the sperm tail response to hypo-osmotic stress according to WHO guidelines (29) are illustrated in Fig. 3. There were significant positive correlations between sperm morphology, motility, concentration and percentage of hypo-osmotic swelling score. The parameter of sperm morphology had a higher significant correlation with HOST values compared to others (P<0.001, Table 2). Also, there was a negative relationship between SDF rate and percentage of HOST (r= -0.50; P<0.001).

**Fig. 4:**
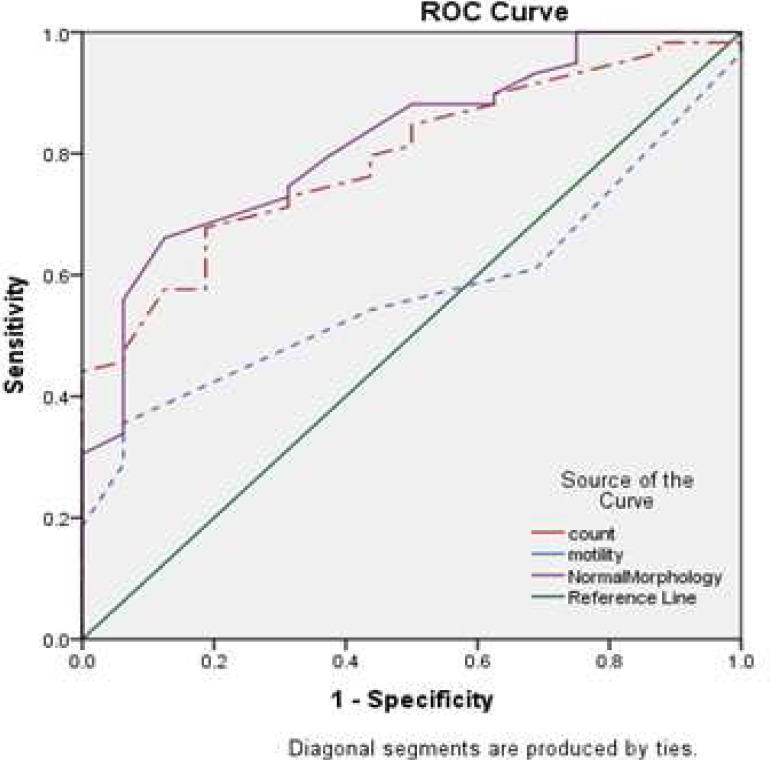
Receiver operating characteristic (ROC) curve analysis for prediction of HOST by sperm parameters. The area under the curve (ACU) for normal morphology, motility and count were 0.81, 0.57 and 0.79, respectively

To evaluate the values of sperm parameters as predictors for HOST, the ROC curve was illustrated (Fig. 4). Diagnostic values and different cut off points of morphology, motility and concentration for prediction of SDF and HOST are shown in Table 3, 4. Among sperm parameters, morphology was the best predictor because it had an accuracy of 0.74 and 0.81 for DF rate and percentage of HOST, respectively (Fig. 2, 4). 

**Table 1:** Relationship between semen parameters and SDF with regression model

**Parameter**	**R²**	**Coefficients(r)**	p-** Value**
Morphology	0.36	0.60	<0.001*
Motility		-0.15	0.10
Concentration		-0.03	0.90

* p- Value< 0.05 was considered significant

**Table 2:** Relationship between semen parameters and HOST

**Parameter**	**R²**	**Coefficients(r)**	p-**Value**
Morphology	0.19	+0.44	<0.001*
Motility		+0.19	0.05*
Concentration		+0.32	0.003*

* p- Value< 0.05 was considered significant

**Table 3:** Sensitivity, specificity and diagnostic values of sperm parameters for the prediction of SDF

	**Cut off point**	**Sensitivity**	**Specificity**	**PPV**	**NPV**
Morphology(%)	4.5	46.7%	80%	78%	50%
Motility(%)	65	17.8%	90%	73%	42%
Concentration(million/ml)	35.5	42%	70%	68%	45%

PPV: positive predictive value; NPV: negative predictive value.

**Table 4:** Sensitivity, specificity and diagnostic values of sperm parameters for the prediction of HOST

	**Cut off point**	**Sensitivity**	**Specificity**	**PPV**	**NPV**
Morphology(%)	5.5	66%	94%	96%	31%
Motility(%)	72.5	29%	94%	94%	26%
Concentration(million/ml)	35.5	46%	94%	96%	32%

PPV: positive predictive value; NPV: negative predictive value

## Discussion

Routine semen analysis is the first line of sperm evaluation in most fertility clinics. Limitations and insufficient value of this simple test lead to proposition of sperm DNA integrity (31) and HOST (32, 33). There are some debates over the necessity of DF test incorporation in male infertility work-up (34, 35). This study attempted to clarify the requirement of these functional tests routinely in IVF clinics.

The distribution of levels of DF in different sperm parameters categories were as follows; in half of samples with 5-15% normal morphology, DF was <30%, while in <5% category it reached up to 60%. The distribution of normal HOST value (≥60%) in category of >15% normal morphology was higher than the others (Fig. 1).

In the present study, there was a significant negative correlation between sperm morphology and DF rate. The correlation between sperm parameters and HOST was positive. There are some reports that indicate a negative correlation between sperm parameters and DF rate (18, 19, 36). Irvin et al. showed that DF rate in semen samples with abnormal morphology and weak (low) sperm motility was higher than normal semen samples (37). Muratori et al. observed no correlation between sperm concentration and DF rate which is similar to our findings (38). Oosterhius et al. used TUNEL assay and reported that sperm concentration was lower in semen samples with high DNA fragmentation Index (DFI) (39). Mehdi et al. reported that DF rate was higher in samples with abnormal morphology but did not find a significant correlation between sperm motility and DF rate (40). Also, in two other studies, there were no relationships between DF rate and sperm parameters (20, 21).

Our findings show significant positive correlation between sperm parameters with percentage of HOST. Some studies reported strong positive correlation between sperm motility, the motile sperm concentration and the percentage of swollen sperms (22-24). Castro et al. observed a weak significant correlation between sperm motility, concentration and HOST (25). Hauser et al. observed no correlation between HOST values and sperm parameters in fresh or thawed sperm (26). AL-Mogazy et al. reported that HOST had asignificant positive correlation with motility, and a negative correlation with count (27).

Stanger and Moskovtsev et al. declared a strong negative correlation between HOST and DF rate. Their findings showed that samples with normal HOST range (>60%) had the least DF rate (28, 41). Also, Oosterhius et al. reported a non-significant negative correlation between HOST and DF rate (39). In Erenpreiss et al.’s report, sperm morphology and motility were main parameters for the prediction of DF rate (42). 

In the present study concerning sperm DF and HOST, the best cut off points for sperm parameters were verified. We also confirmed which sperm parameter(s) is (are) valuable as predictor(s) of DF rate and normal range of HOST. According to our findings, morphology is the best predictor of sperm DF rate and HOST. In men whose sperm normal morphology is <5%, the DF≥30% and HOST< 60%would be expected. In a study, sperm morphology and motility were reported as main parameters for prediction of DF rate (36). In the present report, morphology is the best predictor not only for sperm DF rate but also for HOST.

In conclusion, there are relationships between DF and HOST values with sperm parameters. Sperm parameters have significant correlations with HOST values. Sperm morphology has significant correlation with DF rate and is a predictor for this test. It is therefore not essential to perform sperm parameters, DF and HOST together in all cases of male infertility work-up. The cut off points, significance of correlation, and accuracy of sperm parameters values in relation to DF and HOST are worthy of further consideration.
